# Successful Treatment of Bulla with Endobronchial Valves

**DOI:** 10.1155/2015/947403

**Published:** 2015-08-12

**Authors:** Erdoğan Çetinkaya, Mehmet Akif Özgül, Şule Gül, Hilal Boyacı, Ertan Cam, Emine Kamiloglu, Mustafa Çörtük

**Affiliations:** ^1^Department of Chest Diseases, Yedikule Chest Disease and Chest Surgery Education and Research Hospital, İstanbul, Turkey; ^2^Amasya Merzifon Kara Mustafa Pasa State Hospital, Amasya, Turkey; ^3^Dr. Burhan Nalbantoğlu State Hospital, Lefkose, Northern Cyprus, Turkey; ^4^Karabük University Education and Research Hospital, Karabuk, Turkey

## Abstract

Emphysematous bullae are a complication of end-stage COPD. Patients with large bullae and poor respiratory function have limited treatment options. Surgical resection is a recognized treatment, but functional improvement after bullectomy is not satisfactory in patients with forced expiratory volume in 1 s (FEV1) < 35% predicted. When this 59-year-old male end-stage COPD patient was assessed, he was cachectic and lung function tests showed a FEV1 of 0.56 L (19% predicted) and a RV of 7 L (314% predicted), while 6MWT was 315 m and MRC dyspnea score was 4. Chest X-ray revealed a massive bulla of 10 cm in diameter in the right middle lobe. A fibrobronchoscopy was performed under local anesthesia and 2 Zephyr 4.0 valves were placed in the right middle lobe. Chest X-ray and CT scan performed 36 days later showed the complete resolution of the bulla. Seven months later, the patient demonstrated an improvement in FEV1 (+30%) and a decrease in RV from 314 to 262% predicted. This case report shows that the Zephyr valves may be successfully used to treat a large bulla in the right middle lobe in a patient with diffuse emphysema and severely impaired lung function.

## 1. Introduction

Emphysema is characterized by parenchymal lung destruction and loss of elastic recoil which results in hyperinflation. Bullae are thought to develop from a parenchymal weakness which, once it exceeds a certain size, will result in a space within the lung which will fill preferentially. The bulla does not participate in ventilation to any great extent because of the large volume. Similarly gas exchange is reduced because of the relatively reduced, avascular surface area [1]. Either due to compression of the surrounding lung tissue or simply due to loss of elastic recoil in adjacent airways, there will be occlusion and atelectasis of lung tissue adjacent to the bulla.

Whilst surgical resection of the bulla is a recognized treatment, it has been shown that functional improvement after bullectomy is unsatisfactory in patients with poor respiratory reserve [2].

We report here a case of successful treatment of a large bulla in a patient with poor pulmonary function who had limited treatment options.

## 2. Case Report

A 59-year-old male patient was referred to our center for the assessment of his end-stage chronic obstructive airways disease.

He was an ex-smoker having smoked one pack per day for 40 years until two and a half years prior to the consultation. He had a long history of cough and shortness of breath and was diagnosed with COPD two years earlier. He had worked as a farmer but his worsening COPD had caused him to discontinue his occupation. He was unable to dress or wash himself without assistance, had shortness of breath on minimal exertion, and as a result had become bedridden. Despite maximum use of bronchodilators, inhaled steroids, and long-term oxygen therapy he suffered frequent exacerbations of his COPD requiring hospital admissions. He had no other associated comorbidities and no family history of note.

When assessed in our unit for the first time, he was cachectic with a BMI of 17.9. Blood gases on air revealed a PaO2 of 54.9 torr and PaCO2 of 34 torr. Pulmonary function tests showed a forced expiratory volume in 1 s (FEV1) of 0.56 liters (19% predicted normal value). He had severe hyperinflation with a residual volume (RV) of 7 liters (314% predicted). His 6-minute walk test (6MWT) was 315 meters. His MRC dyspnea score was 4 and lowest oxygen saturation was 76% at rest.

**Figure 1:**
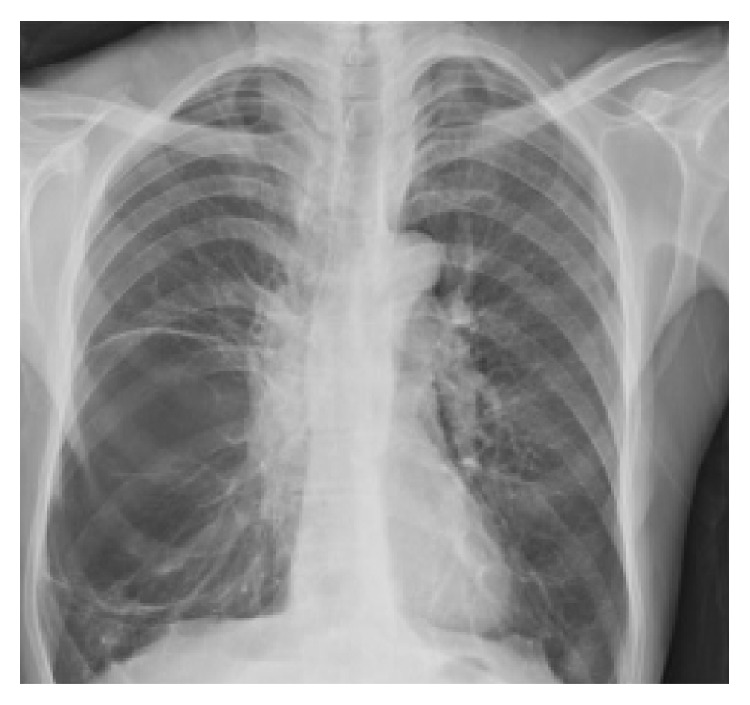
Pretreatment chest X-ray.

**Figure 2:**
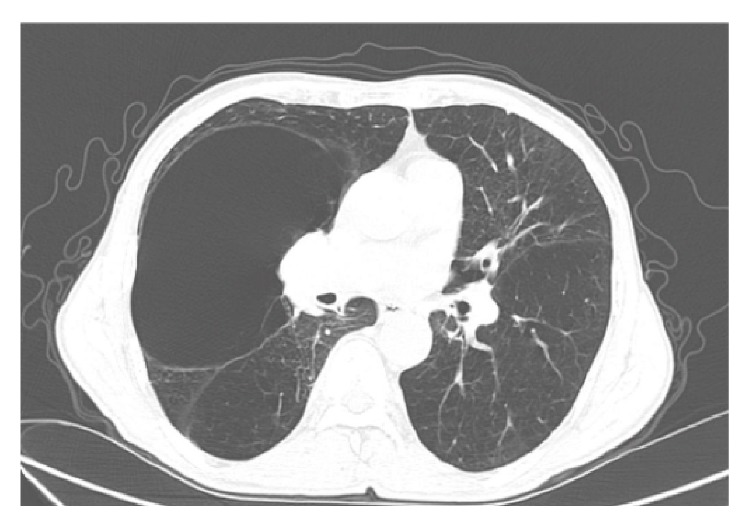
Pretreatment CT scan.

Chest X-ray revealed a bilateral flattening of the diaphragm and bilateral hyperinflation. There was a massive bulla of approximately 10 cm in diameter occupying the right middle lobe (Figure 1). The CT scan confirmed bilateral emphysema and the giant bulla (166 × 85 mm diameter) in the right middle lobe causing compression atelectasis of the right middle and lower lobes (Figure 2). SPECT/CT showed a gross defect in ventilation and perfusion in the area of the right upper and middle lobes.

The patient was assessed for surgical bullectomy. However, it was considered that he would not be a good candidate for surgery in view of the severity of his COPD with the associated poor pulmonary function. Additionally, surgical bullectomy is associated with a significant risk of prolonged postoperative air leak which the patient would not be able to tolerate. After discussion with the patient, it was decided to place one-way endobronchial valves in an attempt to collapse the bulla and allow surrounding lung tissue to expand again.

A fiber-optic bronchoscopy was performed under local anesthesia using lidocaine with the patient under conscious sedation with midazolam. Two Zephyr 4.0 valves were placed in the right middle lobe at B4 and B5. Postoperative chest X-ray did not show any evidence of pneumothorax. The patient made an uneventful recovery and was discharged after a two-day hospital stay.

**Figure 3:**
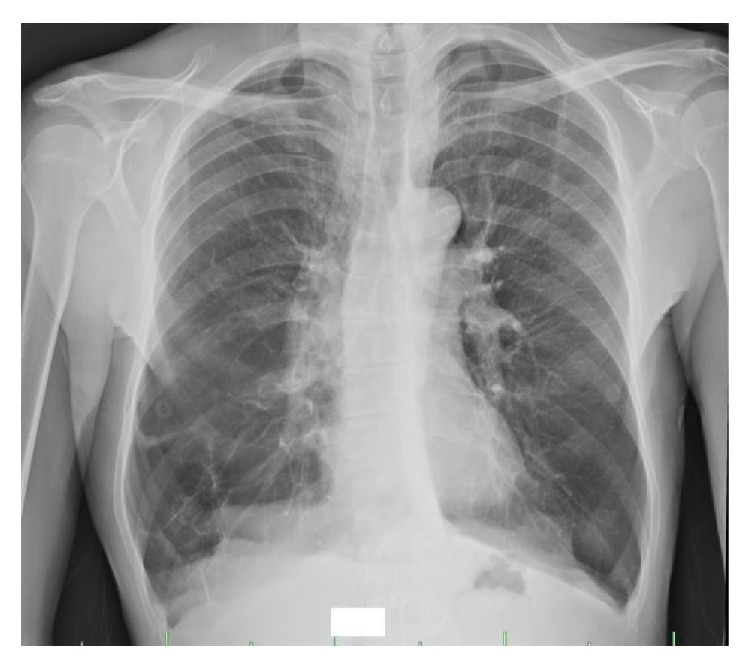
Posttreatment chest X-ray.

**Figure 4:**
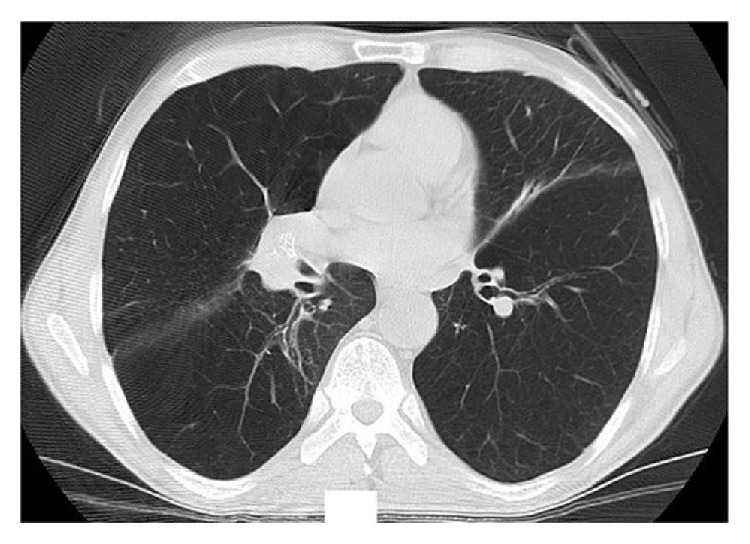
Posttreatment CT scan.

Chest X-ray and CT scan performed 36 days after the procedure showed complete resolution of the right middle lobe bulla (Figures 3 and 4).

Seven months later, the patient demonstrated an improvement in 6MWT of 39% (437 meters). His FEV1 had improved by 30% to 0.73 liters (24% predicted). His RV has reduced from 314% predicted to 262% predicted. He was able to dress himself without help and to travel independently. At this time, he required oxygen supplementation only during exercise. His lowest oxygen saturation has increased 90% at rest.

Additional follow-up data are given in Table 1.

## 3. Discussion

Patients with large bullae and poor respiratory function have limited treatment options. Medical management is limited and whilst surgical resection of the bulla is a recognized treatment, it has been shown that functional improvement after bullectomy is unsatisfactory in patients who have an FEV1 < 35% predicted, severe hypercapnia, and hypoxia [2]. Mortality in patients with diffuse emphysema who had underwent surgical resection of the bulla is higher than in those who did not have diffuse emphysema [3].

Less invasive approaches for patients with poor respiratory cell reserve have been considered. Takizawa et al. used CT guided drainage but prolonged air leak was a significant problem [4]. Bhattacharyya et al. decompressed an emphysematous bulla via a transbronchial aspiration needle and instilled autologous blood into the bulla to induce fibrosis. However, the risk of pneumothorax and bronchopleural fistula makes this procedure unsuitable for peripheral bullae [5].

The Zephyr Endobronchial Valve (Zephyr EBV) is designed to create volume reduction in patients with hyperinflation associated with emphysema. The device consists of a one-way, silicone, duckbill valve attached to a nickel-titanium (Nitinol), self-expanding retainer that is covered with a silicone membrane. It is implanted in the target bronchus using a flexible delivery catheter that is guided to the targeted bronchus by inserting it through a 2.8 mm working channel of a bronchoscope. It allows air and secretions to escape from the occluded lobe on expiration but prevents air from entering on inspiration [6].

For the treatment of a bulla, by placing EBV in the airways communicating with the bulla, air is able to escape from the bulla on expiration but no air enters on inspiration and, as a result, the bulla deflates. Respiratory function should improve either as a result of less compression of the surrounding tissue or simply as a result of the elimination of dead space allowing better ventilation and breathing mechanics. The EBVs are designed to be a permanent implant. However, in the event that there is no positive benefit or an adverse effect, the EBVs can easily be removed via a bronchoscope.

Santini et al. have reported a series of nine patients who benefited from EBV placement for the treatment of giant bullae. Mean FEV1 was 1.0 (35% predicted) and mean RV was 5.5 liters (231% predicted) [7]. In the case reported here, the patient was severely compromised with a FEV1 of 0.56 liters (19% predicted) and a RV of 7 liters (314% predicted), but treatment was successful with resolution of the bulla, improved pulmonary function tests, and improved performance with an increase in 6MWT and the ability to recommence daily life activities.

In the Santini's series, the bullae were situated in the left upper lobe in five patients, right upper lobe in two patients, left lower lobe in one patient, and bilateral upper lobes in one patient [7]. Fiorelli et al. have published 15 patients with emphysema and 10 patients have isolated giant bullous. They found improvement in FEV1 for patient who has giant bullous higher than who has emphysema (11.7% versus 7%) [8]. In this study, occupation of the giant bulla in the chest, as well as compression of surrounding tissue and diaphragm, therefore after EBV applying that reported improvement in lung function. In our case, the bulla was situated in the right middle lobe and was successfully treated. It is not certain what role collateral ventilation has in maintaining the structure of bullae. However, when EBVs are used to create lung volume reduction in emphysema, superior results are obtained in the absence of collateral ventilation. Raasch et al. found that the fissures between the right middle lobe and adjacent lobes were incomplete in a high proportion of cases (94% minor fissure, 70% right upper major, and 47% right lower major), suggesting a high likelihood of collateral ventilation between right middle and adjacent lobes [9]. Success in this case may be explained because the placement of EBV blocked all airways feeding the bulla or, if additional channels were present, the resistance may have been sufficiently high that air preferentially escaped via the EBV, thus reducing the size of the bulla and increasing the resistance of collateral channels further.

## 4. Conclusion

In this case, the Zephyr Endobronchial Valves were successfully used to treat a large bulla in the right middle lobe in a patient with diffuse emphysema and severely impaired pulmonary function. Although a single case report, this case provides additional evidence to the case series of Santini et al. that EBV can be used to offer a safe treatment option for patients with emphysematous bullae who are not good candidates for surgery.

## Figures and Tables

**Table 1:** Investigation results before treatment and at 7 months and 17 months after treatment.

	Before treatment (% predicted)	7 months after treatment (% predicted)	17 months after treatment (% predicted)
6MWT (m)	315	437	378
Pulmonary function tests			
FVC (L)	2.05 (54)	2.76 (73)	2.43 (65)
FEV1 (L)	0.56 (19)	0.73 (24)	0.61 (20)
IC (L)	0.83 (29)	1.42 (49)	1.20 (41)
TLC (L)	9.14 (146)	8.79 (140)	8.70 (139)
RV (L)	7.00 (314)	5.86 (262)	6.02 (267)
MRC dyspnea score	4	3	Not available
